# Role of exosome-associated adenosine in promoting angiogenesis

**DOI:** 10.20517/2574-1209.2019.37

**Published:** 2020-04-10

**Authors:** Nils Ludwig, Edwin K. Jackson, Theresa L. Whiteside

**Affiliations:** 1 Department of Pathology, University of Pittsburgh School of Medicine, Pittsburgh, PA 15213, USA; 2 UPMC Hillman Cancer Center, Pittsburgh, PA 15213, USA; 3 Department of Pharmacology and Chemical Biology, University of Pittsburgh School of Medicine, Pittsburgh, PA 15213, USA; 4 Departments of Immunology and Otolaryngology, University of Pittsburgh School of Medicine, Pittsburgh, PA 15213, USA

**Keywords:** Exosomes, extracellular vesicles, angiogenesis, adenosine, adenosine receptors, endothelial cells

## Abstract

The role of exosomes in different physiological and pathological settings is an emerging field of great current interest. One hallmark of exosomes is the promotion of blood vessel formation. Exosomes of different cellular origin have been shown to be enriched in angiogenic proteins which directly promote angiogenesis. In addition, exosomes are also efficacious producers of adenosine and potentially encapsulate adenosine in their lumen. The adenosine content of exosomes has been linked to their immunosuppressive effects. In this communication, we consider the possibility that adenosine production by tumor cell-derived exosomes may represent a novel pathway for stimulation of angiogenesis in the tumor microenvironment.

## INTRODUCTION

Extracellular vesicles (EVs) and their functional role in health and disease are of great current interest. Especially exosomes, a virus-size subset of EVs (~30–100 nm), show great potential as disease biomarkers, drug carriers, or therapeutics. They are actively produced by parent cells and carry a complex cargo, which includes proteins, nucleic acids, and lipids^[1]^.

## ROLES OF EXOSOMES IN ANGIOGENESIS

One hallmark of exosomes is the promotion of angiogenesis. Skog *et al.*^[2]^ reported in 2008 that glioblastoma-derived exosomes contain mRNA, microRNA (miRNA), and angiogenic proteins and that these exosomes reprogram endothelial cells (ECs) to an angiogenic phenotype. A variety of research groups extended the work of Skog *et al.*^[2]^ addressing the pro-angiogenic functions of exosomes in different health and disease settings. Multiple pathways which are used by exosomes to stimulate blood vessel formation were uncovered in recent years and most research focuses on the direct interaction of exosomes with ECs^[3]^. It was shown that exosomes can deliver signals to receptors on ECs, which activate the relevant molecular pathways and contribute to altered cellular responses^[3]^. The reported ligands on the surface of exosomes which can induce an angiogenic response include vascular endothelial growth factor (VEGF), interleukin 6 (IL-6), IL-8, fibroblast growth factors (FGF), and urokinase-type plasminogen activator (uPA)^[2,4]^. Besides surface-mediated receptor-ligand interactions, ECs internalize exosomes within 2–4 h^[4]^. Numerous pathways are utilized by ECs for the internalization of exosomes, such as phagocytosis, micropinocytosis, or lipid raft-mediated internalization^[5]^. However, the main uptake mechanism is endocytosis, as recently reported^[4]^. The internalization of exosomes allows for the delivery of messages that are then translated by ECs^[2]^. miRNAs are frequently described components of the exosome cargo which can induce pro-angiogenic responses in ECs^[6]^.

## ADENOSINE PATHWAY AND ANGIOGENESIS

In addition to these well-described pathways, signaling via purines may also contribute to exosome-mediated effects on angiogenesis. One pathway that has not been studied thus far and that is particularly interesting in the context of exosomes and angiogenesis is the adenosine pathway. Extracellular adenosine exhibits a broad range of effects on cell cycle control, immunoregulation, and cytokine regulation through both direct and indirect mechanisms and ultimately leads to the progression of malignant diseases^[7]^. Additionally, adenosine has been recognized as a potent stimulator of angiogenesis and Adair estimated that adenosine can contribute up to 50%−70% of the angiogenic response in some situations^[8,9]^. Adair *et al.*^[10]^ also described that intravenous infusion of adenosine can increase plasma levels of VEGF in humans. In cultured cells, it was shown that adenosine induces EC proliferation and migration by increasing levels of VEGF and other angiogenic growth factors^[9]^. Additionally, adenosine can stimulate EC proliferation independently of VEGF, which probably involves modulation of other pro-angiogenic and anti-angiogenic growth factors and perhaps an intracellular mechanism^[9]^.

Initiation of the adenosine signaling cascade requires binding of adenosine to the specific adenosine receptors (ADORs), which are divided into four subtypes: A_1_R, A_2A_R, A_2B_R and A_3_R. The main difference between these receptors is the affinity for adenosine, since adenosine binds to A_1_R, A_2A_R and A_3_R in the nanomolar range, whereas adenosine binds to A_2B_R in the micromolar range^[11]^. This indicates that physiologic concentrations are sufficient to induce A_1_R-, A_2A_R- and A_3_R-mediated signaling, and elevated adenosine concentrations, which are usually found in inflammatory or tumor microenvironments, can activate an A_2B_Rsignaling cascade^[11]^. It was shown that adenosine induces EC growth by activating A_2B_R and that A_2B_R plays a critical role in regulating vascular remodeling associated with EC proliferation in angiogenesis, collateral vessel development, and recovery after vascular injury^[12,13]^. However, the other receptor subtypes have also been reported to be involved in angiogenesis. Although there is no direct stimulation of ECs, ADORs act in a functional cooperative fashion to promote angiogenesis by a paracrine mechanism involving the differential expression and secretion of angiogenic factors from other cell types^[14,15]^. Ernens *et al.*^[16]^ reported that adenosine upregulates thrombospondin-1 production by macrophages via A_2A_R and A_2B_R, resulting in stimulation of angiogenesis. Adenosine also stimulates the production of VEGF, IL-8, and angiopoetin-1 from mast cells via A_2B_R and A_3_R, as reported by Feoktistov *et al.*^[15]^. Clark *et al.*^[17]^ demonstrated that A_1_R activation elicits an angiogenic response and promotes VEGF-release from cultured monocytes. Thus, the literature suggests that all ADORs are involved in regulating blood vessel development, and that the underlying pathway for stimulating angiogenesis is highly context/microenvironment-dependent.

## ADENOSINE-MEDIATED STIMULATION OF ANGIOGENESIS BY EXOSOMES

**Figure 1.**
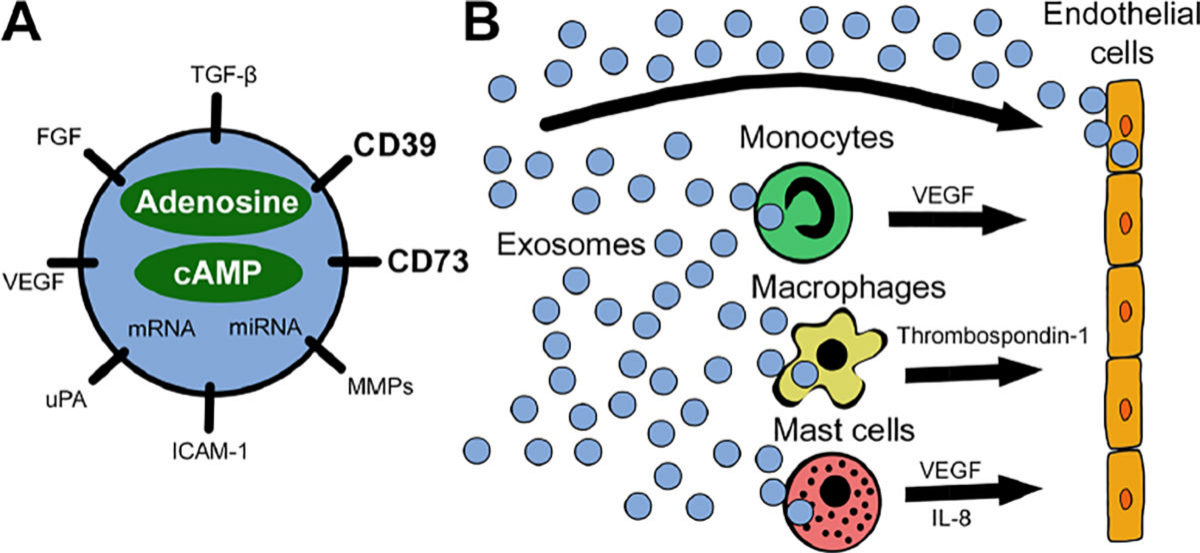
A schematic visualizing the reprogramming of endothelial cells by exosomes. A: exosomes carry a variety of pro-angiogenic factors including the ectonucleotidases CD39 and CD73. Besides surface-bound molecules, exosomes encapsulate pro-angiogenic factors, nucleic acids, adenosine, and cAMP, as well as other purine metabolites; B: tumor-derived exosomes interact directly with endothelial cells or reprogram other cells in the tumor microenvironment to release pro-angiogenic factors. All these interactions involve signaling via adenosine receptors expressed on responder cells: specifically, A_2B_R on endothelial cells, A_1_R on monocytes, A_2A_R and A_2B_R on macrophages, and A_2B_R and A_3_R on mast cells. miRNA: microRNA; VEGF: vascular endothelial growth factor; FGF: fibroblast growth factor; TGF-β: transforming growth factor beta; MMPs: matrix metalloproteinases; ICAM-1: intercellular adhesion molecule 1; uPA: urokinase-type plasminogen activator; IL-8: interleukin 8; cAMP: cyclic adenosine monophosphate

Taken together, the above findings suggest the presence of a possible link between angiogenesis and exosome-associated adenosine, as presented in Figure 1. Specifically, it was shown that exosomes contribute to extracellular adenosine production and hence might modulate ECs indirectly^[18]^. Exosomes from diverse cancer cell types exhibit potent ATP- and 5’-AMP-phosphohydrolytic activity, partly attributed to activity of CD39 and CD73, respectively, on the surface of exosomes^[19]^. This exosome-generated adenosine is functionally active and can trigger a cyclic adenosine monophosphate (cAMP) response in A_2A_R-positive but not A_2A_R-negative cells^[19]^.

While it is well recognized that exosomes encapsulate functional proteins and nucleic acids, it is currently unclear whether purine metabolites are encapsulated within exosomes. Sayner *et al.*^[20]^ recently reported that EVs encapsulate cAMP to provide an additional second messenger compartment. Our preliminary data show that exosomes not only encapsulate cAMP but also adenosine and adenosine metabolites (inosine, hypoxanthine, and xanthine). This may indicate that exosomes can induce ADOR signaling independently of the production of adenosine by exosome-associated enzymes. Adenosine in the lumen of exosomes is protected against uptake and metabolism by other cells, such as red blood cells. Thus, exosomal adenosine may represent a mechanism for adenosine to serve as a circulating, rather than strictly local, factor.

Exosome-associated adenosine emerges as a potential stimulator of angiogenesis in different settings. Studying this pathway promises to uncover important aspects of exosome functions, which are ultimately leading to the stimulation of blood vessel formation. Understanding this pathway might also help to find targets for the stimulation or inhibition of exosome-induced angiogenesis.

## References

[1] Whiteside TL. Tumor-derived exosomes and their role in cancer progression. Adv Clin Chem 2016;74:103–41. 27117662 10.1016/bs.acc.2015.12.005 PMC5382933

[2] Skog J, Würdinger T, van Rijn S, Meijer DH, Gainche L, Glioblastoma microvesicles transport RNA and proteins that promote tumour growth and provide diagnostic biomarkers. Nat Cell Biol 2008;10:1470–6. 19011622 10.1038/ncb1800 PMC3423894

[3] Ludwig N, Whiteside TL. Potential roles of tumor-derived exosomes in angiogenesis. Expert Opin Ther Targets 2018;22:409–17. 29634426 10.1080/14728222.2018.1464141 PMC6126896

[4] Ludwig N, Yerneni SS, Razzo BM, Whiteside TL. Exosomes from HNSCC promote angiogenesis through reprogramming of endothelial cells. Mol Cancer Res 2018;16:1798–808. 30042174 10.1158/1541-7786.MCR-18-0358

[5] Mulcahy LA, Pink RC, Carter DR. Routes and mechanisms of extracellular vesicle uptake. J Extracell Vesicles 2014;3. 10.3402/jev.v3.24641 PMC4122821 25143819

[6] Umezu T, Ohyashiki K, Kuroda M, Ohyashiki JH. Leukemia cell to endothelial cell communication via exosomal miRNAs. Oncogene 2013;32:2747–55. 22797057 10.1038/onc.2012.295

[7] Cai Y, Feng L, Wang X. Targeting the tumor promoting effects of adenosine in chronic lymphocytic leukemia. Crit Rev Oncol Hematol 2018;126:24–31. 29759563 10.1016/j.critrevonc.2018.03.022

[8] Ethier MF, Chander V, Dobson JG Jr. Adenosine stimulates proliferation of human endothelial cells in culture. Am J Physiol 1993;265:H131–8. 8342624 10.1152/ajpheart.1993.265.1.H131

[9] Adair TH. Growth regulation of the vascular system: an emerging role for adenosine. Am J Physiol Regul Integr Comp Physiol 2005;289:R283–96. 16014444 10.1152/ajpregu.00840.2004

[10] Adair TH, Cotten R, Gu JW, Pryor JS, Bennett KR, Adenosine infusion increases plasma levels of VEGF in humans. BMC Physiol 2005;5:10. 15967042 10.1186/1472-6793-5-10 PMC1183224

[11] Azambuja JH, Ludwig N, Braganhol E, Whiteside TL. Inhibition of the Adenosinergic Pathway in Cancer Rejuvenates Innate and Adaptive Immunity. Int J Mol Sci 2019;20:5698. 10.3390/ijms20225698 PMC6888217 31739402

[12] Dubey RK, Gillespie DG, Jackson EK. A(2B) adenosine receptors stimulate growth of porcine and rat arterial endothelial cells. Hypertension 2002;39:530–5. 11882603 10.1161/hy0202.103075

[13] Du X, Ou X, Song T, Zhang W, Cong F, Adenosine A2B receptor stimulates angiogenesis by inducing VEGF and eNOS in human microvascular endothelial cells. Exp Biol Med (Maywood) 2015;240:1472–9. 25966978 10.1177/1535370215584939 PMC4935298

[14] Auchampach JA. Adenosine receptors and angiogenesis. Circ Res 2007;101:1075–7. 18040023 10.1161/CIRCRESAHA.107.165761 PMC2399891

[15] Feoktistov I, Ryzhov S, Goldstein AE, Biaggioni I. Mast cell-mediated stimulation of angiogenesis: cooperative interaction between A2B and A3 adenosine receptors. Circ Res 2003;92:485–92. 12600879 10.1161/01.RES.0000061572.10929.2D

[16] Ernens I, Bousquenaud M, Lenoir B, Devaux Y, Wagner DR. Adenosine stimulates angiogenesis by up-regulating production of thrombospondin-1 by macrophages. J Leukoc Biol 2015;97:9–18. 25387836 10.1189/jlb.3HI0514-249RR

[17] Clark AN, Youkey R, Liu X, Jia L, Blatt R, A1 adenosine receptor activation promotes angiogenesis and release of VEGF from monocytes. Circ Res 2007;101:1130–8. 17901362 10.1161/CIRCRESAHA.107.150110

[18] Schuler PJ, Saze Z, Hong CS, Muller L, Gillespie DG, Human CD4+ CD39+ regulatory T cells produce adenosine upon coexpression of surface CD73 or contact with CD73+ exosomes or CD73+ cells. Clin Exp Immunol 2014;177:531–43. 24749746 10.1111/cei.12354 PMC4226604

[19] Clayton A, Al-Taei S, Webber J, Mason MD, Tabi Z. Cancer exosomes express CD39 and CD73, which suppress T cells through adenosine production. J Immunol 2011;187:676–83. 21677139 10.4049/jimmunol.1003884

[20] Sayner SL, Choi CS, Maulucci ME, Ramila KC, Zhou C, Extracellular vesicles: another compartment for the second messenger, cyclic adenosine monophosphate. Am J Physiol Lung Cell Mol Physiol 2019;316:L691–700. 30758991 10.1152/ajplung.00282.2018 PMC6483015

